# Depression Screening and Patient Outcomes in Cancer: A Systematic Review

**DOI:** 10.1371/journal.pone.0027181

**Published:** 2011-11-14

**Authors:** Anna Meijer, Michelle Roseman, Katherine Milette, James C. Coyne, Michael E. Stefanek, Roy C. Ziegelstein, Erin Arthurs, Allison Leavens, Steven C. Palmer, Donna E. Stewart, Peter de Jonge, Brett D. Thombs

**Affiliations:** 1 Interdisciplinary Center for Psychiatric Epidemiology, University Medical Center Groningen, University of Groningen, Groningen, The Netherlands; 2 Lady Davis Institute for Medical Research, Jewish General Hospital, Montréal, Québec, Canada; 3 Department of Psychiatry, McGill University, Montréal, Quebéc, Canada; 4 Department of Educational and Counselling Psychology, McGill University, Montréal, Quebéc, Canada; 5 Department of Epidemiology, Biostatistics, and Occupational Health, McGill University, Montréal, Quebéc, Canada; 6 Department of Medicine, McGill University, Montréal, Quebéc, Canada; 7 Behavioral Oncology Program, Abramson Cancer Center and Department of Psychiatry, University of Pennsylvania School of Medicine, Philadelphia, Pennsylvania, United States of America; 8 Health Psychology Section, Department of Health Sciences, University Medical Center Groningen, University of Groningen, Groningen, The Netherlands; 9 Office of Research Administration, Indiana University, Bloomington, Indiana, United States of America; 10 Department of Medicine, Johns Hopkins University School of Medicine, Baltimore, Maryland, United States of America; 11 LIVESTRONG Survivorship Center of Excellence, Cancer Control, and Outcomes, Abramson Cancer Center, University of Pennsylvania, Philadelphia, Pennsylvania, United States of America; 12 Women's Health Program, University Health Network, Toronto, Ontario, Canada; 13 Departments of Psychiatry, Obstetrics and Gynaecology, Family and Community Medicine, Medicine, Surgery and Anesthesia, University of Toronto, Toronto, Ontario, Canada; Federal University of Rio de Janeiro, Brazil

## Abstract

**Background:**

Several practice guidelines recommend screening for depression in cancer care, but no systematic reviews have examined whether there is evidence that depression screening benefits cancer patients. The objective was to evaluate the potential benefits of depression screening in cancer patients by assessing the (1) accuracy of depression screening tools; (2) effectiveness of depression treatment; and (3) effect of depression screening, either alone or in the context of comprehensive depression care, on depression outcomes.

**Methods:**

Data sources were CINAHL, Cochrane, EMBASE, ISI, MEDLINE, PsycINFO and SCOPUS databases through January 24, 2011; manual journal searches; reference lists; citation tracking; trial registry reviews. Articles on cancer patients were included if they (1) compared a depression screening instrument to a valid criterion for major depressive disorder (MDD); (2) compared depression treatment with placebo or usual care in a randomized controlled trial (RCT); (3) assessed the effect of screening on depression outcomes in a RCT.

**Results:**

There were 19 studies of screening accuracy, 1 MDD treatment RCT, but no RCTs that investigated effects of screening on depression outcomes. Screening accuracy studies generally had small sample sizes (median = 17 depression cases) and used exploratory methods to set sample-specific cutoff scores that varied substantially across studies. A nurse-delivered intervention for MDD reduced depressive symptoms moderately (effect size = 0.37).

**Conclusions:**

The one treatment study reviewed reported modest improvement in depressive symptoms, but no evidence was found on whether or not depression screening in cancer patients, either alone or in the context of optimal depression care, improves depression outcomes compared to usual care. Depression screening in cancer should be evaluated in a RCT in which all patients identified as depressed, either through screening or via physician recognition and referral in a control group, have access to comprehensive depression care.

## Introduction

Over 40% of people will be diagnosed with cancer in their lifetime with two-thirds living at least 5 years [1], [2]. Cancer treatment is often arduous and may include surgery, radiotherapy, or chemotherapy that can last for months or years. Cancer patients and survivors often experience decreased quality of life, reduced capacity to perform daily activities, and mental health problems. Distress is common, ranging from “normal” distress in reaction to cancer and its treatment to symptoms that meet criteria for a psychiatric disorder [3], [4]. Prevalence of major depressive disorder (MDD) is estimated to be approximately 11% among cancer patients, compared to 5–6% in the general population, although rates may vary depending on the type of cancer [5], [6].

Many cancer patients report that their psychosocial needs are not addressed adequately, and improving supportive and palliative care has been prioritized [3], [4], [7]. A 2002 US National Institutes of Health (NIH) State-of-the Science Conference Statement [8] called for the routine use of screening tools to identify untreated depression among cancer patients. Similarly, among gaps in psychosocial care, a 2007 report from the Institute of Medicine (IOM) noted low rates of recognition and treatment for depression [4]. The IOM report [4] and guidelines from the UK National Institute for Clinical Excellence (NICE) [7] and the National Comprehensive Cancer Network (NCCN) [3] recommend screening for psychological “distress,” including depression, in cancer patients.

The term *screening* has been used, sometimes inaccurately, to describe a number of activities that involve the use of depression symptom questionnaires, including using the questionnaires to monitor symptom severity or treatment effects, to detect relapse in patients who have undergone treatment, to identify patients who are receiving suboptimal treatment, or to inform the delivery of psychosocial services that are provided to all patients, regardless of symptom severity scores. Although these activities are potentially useful applications of depression symptom questionnaires, none constitutes screening [9]. Screening, as defined by the UK National Screening Committee, is “a public health service in which members of a defined population, who do not necessarily perceive they are at risk of, or are already affected by, a disease or its complications, are asked a question or offered a test to identify those individuals who are more likely to be helped than harmed by further tests or treatment to reduce the risk of disease or its complications” (page 6) [10]. Thus, screening for MDD involves using questionnaires to identify patients who may have depression, but who are not seeking treatment for symptoms and whose depression is not otherwise recognized. Patients who screen positive should be further assessed using a clinical interview to determine if a diagnosis of MDD is warranted, and, if appropriate, treated. In addition to evidence from well-designed and conducted screening randomized controlled trials (RCTs), established criteria for when recommendations for screening should be considered [10]–[12] emphasize the need to assess whether accurate screening tests with only a tolerably small risk of false positive results are available and whether there are effective treatments for patients identified through screening.

**Figure 1:**
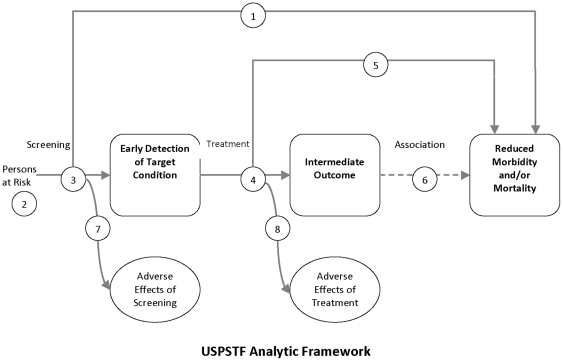
USPSTF Framework for Evaluating Screening Programs.

No systematic reviews have specifically evaluated the effects of screening for MDD in cancer patients on depression outcomes. Thus, the objective of this systematic review was to evaluate whether evidence supports recommendations for systematic screening for depression in cancer care. We used the US Preventive Services Task Force (USPSTF) [13], [14] analytic framework for evaluating evidence for or against screening programs to develop review questions (see Figure 1). The USPSTF framework recognizes the need for RCTs to directly assess links between screening programs and patient outcomes. When direct evidence from RCTs is not available or is of low quality, the USPSTF framework assesses key links that are necessary for screening to benefit patients, focusing on the need for accurate screening tools and effective treatments [14]. Thus, we identified the following key questions for the current review:

**Key Question # 1:** What is the accuracy of depression screening instruments among cancer patients?
**Key Question # 2:** Does treatment of depression improve symptoms of depression in cancer patients?
**Key Question # 3:** Is depression screening of cancer patients, either alone or in the context of enhanced depression care, more effective than usual care in reducing depressive symptoms or diagnoses of MDD?

## Methods

### Search strategy

The CINAHL, Cochrane, EMBASE, ISI, MEDLINE, PsycINFO and SCOPUS databases were searched through January 24, 2011. One search was conducted to identify articles that compared a screening instrument with a valid MDD criterion standard (Key Question #1) or that assessed outcomes from depression screening, either alone or in the context of enhanced depression care (Key Question #3). A second search was done for depression treatment studies (Key Question #2). See Supplementary Information S1 for search terms. Manual searching was done on reference lists of included articles, relevant systematic reviews (Supplementary Information S2), and 45 selected journals (August 2010 to January 2011; Supplementary Information S3). We tracked citations of included articles using Google Scholar [15], surveyed authors of included treatment and screening trials, and searched the trial registries ClinicalTrials.gov [16] and the International Standard Randomized Controlled Trial Number Register [17] to attempt to identify unpublished treatment or screening RCTs.

### Identification of eligible studies

Eligible articles included studies in any language on cancer patients with any type of malignancy at any disease stage that reported original data, excluding case series or case reports. Translators assisted reviewers to evaluate titles/abstracts and articles for languages not covered by investigators, who were able to independently review material in English, Dutch, French, and Spanish. Multiple articles on the same cohort were treated as a single study. Studies with mixed populations were included if cancer data were reported separately.

Studies on the accuracy of depression screening tools (Key Question #1) were included if they compared screening results to a *Diagnostic and Statistical Manual of Mental Disorders (DSM)* or *International Classification of Diseases (ICD)* diagnosis of MDD based on a validated structured or semi-structured interview (e.g., Structured Clinical Interview for DSM-IV [SCID-IV] [18], Composite International Diagnostic Interview [CIDI] [19], Diagnostic Interview Schedule [DIS] [20]) administered within 2 weeks of the screening tool and reporting data allowing determination of sensitivity, specificity, positive predictive value, and negative predictive value.

Eligible articles on depression treatment (Key Question #2) were RCTs comparing pharmacological, psychotherapeutic, or other interventions with placebo or usual care controls among cancer patients diagnosed with MDD based on a validated diagnostic interview and DSM or ICD criteria. We required a valid diagnostic interview because unassisted clinician diagnoses have poor reliability [21] and because a large proportion of patients scoring above cutoffs on self-report questionnaires do not have MDD [22]. Head-to-head trials of different interventions without a comparison to usual care or placebo were not eligible.

Eligible articles for Key Question #3 were RCTs that compared depression outcomes between cancer patients who underwent depression screening and those who did not. We searched for both screening studies that included the provision of comprehensive depression care for patients with depression as part of the screening program and studies that screened patients, but did not provide such care. Changes in rates of depression recognition and treatment were noted, but not included as depression outcomes. This is because increased treatment without improved depression outcomes would expose patients to costs and potential harms without benefit. Screening was defined per the UK National Screening Committee's definition [10]. Thus, eligible screening trials had to include a case identification strategy based on an *a priori* defined cutoff score on a depression screening tool to make decisions regarding further assessment or treatment. Studies in which both intervention and control groups received the same psychosocial services, but service providers in the intervention group had access to results from psychosocial questionnaires that may have informed their interactions, but did not necessarily determine service allocation decisions, were not included. Studies in which questionnaire results were provided to clinicians without guidance on cutoff scores to determine positive screening status were also excluded. Finally, studies that administered multiple screening tools for multiple problems were not included, since determining whether depression screening influenced depression outcomes would not be possible.

Two investigators independently reviewed articles for eligibility. If either deemed an article potentially eligible based on title/abstract review, then a full-text review was completed. Disagreements after full-text review were resolved by consensus.

### Evaluation of eligible studies

Two investigators independently extracted and entered data into a standardized spreadsheet (see Supplementary Information S4). Discrepancies were resolved by consensus. For Key Question #1 (diagnostic accuracy), the Quality Assessment for Diagnostic Accuracy Studies tool (QUADAS) [23] was used for quality assessment (see Supplementary Information S5). Risk of bias in studies included for Key Question #2 (treatment) and Key Question #3 (screening) was assessed with the Cochrane Risk of Bias tool [24] (see Supplementary Information S6). Study quality and risk of bias were assessed by 2 investigators with discrepancies resolved by consensus.

### Data presentation and synthesis

In studies included for Key Question #1 (diagnostic accuracy), for each screening instrument, sensitivity, specificity, positive predictive value, and negative predictive value with 95% confidence intervals (CIs) [25] were extracted based on primary cutoffs identified by study authors. For Key Questions #2 (treatment) and #3 (screening), when multiple depression outcomes were reported, designated primary outcomes for each study were prioritized, followed by observer-rated scales, then self-report measures. Post-intervention effect sizes were reported using the Hedges's *g* statistic [26], which represents a standardized difference between 2 means, as well as *r^2^*, which is statistically equivalent [27], [28], but presents results in terms of percent of variance in depression change scores due to treatment. Response and remission were presented as relative risk ratios using study definitions.

Eligible studies for each key question were evaluated to determine whether there was sufficient clinical and methodological similarity to support pooling of results. For Key Question #1, studies were heterogeneous in terms of patient samples, screening tools and cutoffs, criterion standards, and whether they used *a priori*-defined, standard scoring thresholds versus sample-specific thresholds based on exploratory receiver operating characteristic (ROC) curve methods. Only 1 eligible study was identified for Key Question #2 and none for Key Question #3. Thus, results were not pooled quantitatively.

A review protocol was not published or registered for this study. However, a protocol was followed for searching, data extraction, and data synthesis with all methods determined *a priori*.

## Results

### Key Question #1: Diagnostic Accuracy of Depression Screening Tools

**Figure 2:**
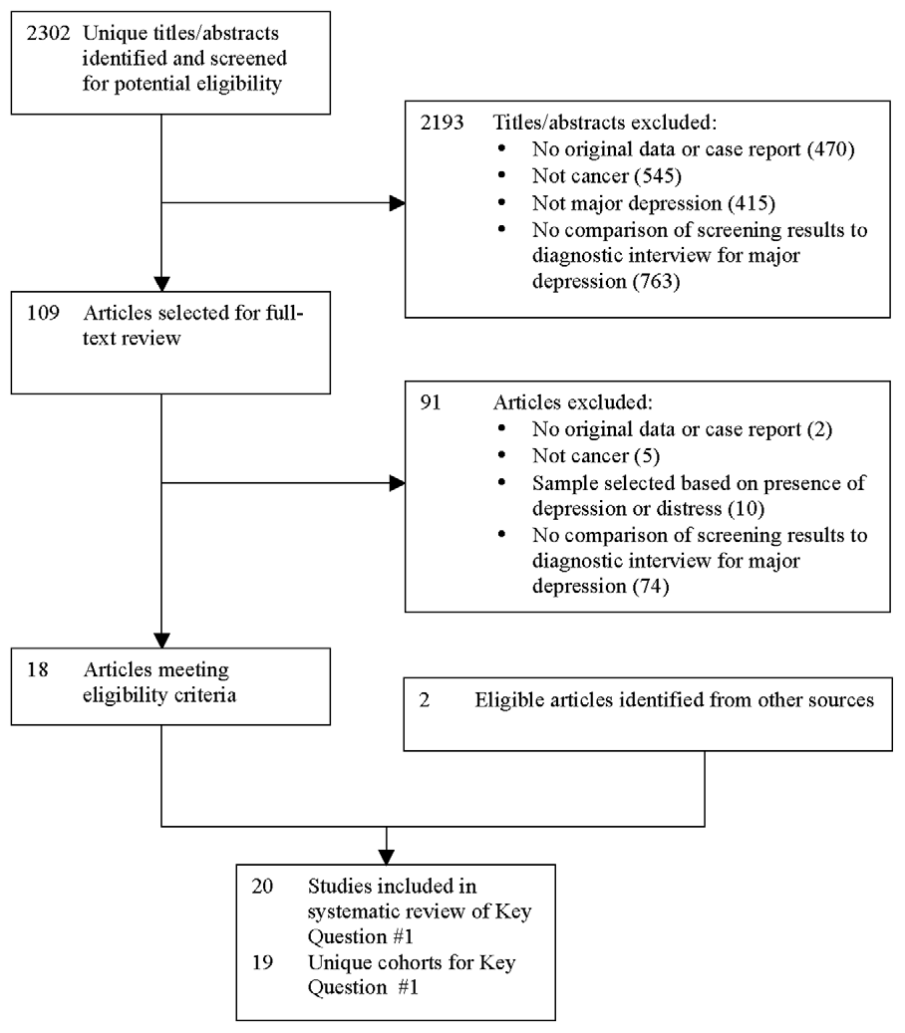
PRISMA Flow Diagram of Study Selection Process for Key Question #1.

The database search for Key Questions #1 (diagnostic accuracy) and #3 (screening) generated 2,302 unique citations (Figure 2). For Key Question #1 (diagnostic accuracy), 2,193 were excluded after title/abstract review and 91 after full-text review. Two additional eligible articles [29], [30] were identified through alternative sources, resulting in 20 included articles [29]–[48]. Two of these articles [37], [38] reported on the same cohort, leaving 19 unique studies for review.

The 19 studies reviewed included 8 studies of breast cancer patients [29], [30], [33], [35], [40], [41], [44], [46] and 11 of patients with mixed cancer sites [31], [32], [34], [36]–[39], [42], [43], [45], [47], [48] across the spectrum of cancer stages (Table 1). Sample sizes in the 19 patient cohorts ranged from 16 to 381 (median = 128), and the number of cases of MDD from 6 to 74 (median = 17). In 12 studies [31]–[39], [41], [44], [47], [48], diagnostic accuracy data were reported using an optimal cutoff score that maximized accuracy based on exploratory ROC methods (Table 2); 1 study [46] used exploratory methods for the study's primary screening tool and compared results to literature-based cutoffs for 2 other screening tools; 1 study [45] used exploratory methods to identify an optimal cutoff among a small set of possible cutoffs from the literature; and 5 studies [29], [30], [40], [42], [43] reported on standard cutoff scores from the screening literature.

**Table 1:** Patient Characteristics in Studies of Diagnostic Accuracy of Depression Screening Tools.

First Author, Year	Country	Cancer Site/Description	N	Mean Age (Years)	Males (%)	N (%) Major Depression
Akechi [31], 2006	Japan	Mixed/Terminal	209	61	66	14 (7%)
Alexander [30], 2010	UK	Breast/Stage I–IIb (disease-free)	200	58	0	18 (9%)
Coyne [29], 2004	USA	Breast/Stage I–IV (Total Sample)^a^	113	56	0	10 (9%)
		Breast/Stage I–IV (Excluding Treated MDD/GAD)^a^	103	56^b^	0	3 (3%)
Grassi [32], 2009	Italy	Mixed/Local, loco-regional, or metastatic	79	57^c^	24^c^	14 (18%)
Hopwood [33], 1991	UK	Breast/Advanced	81	NR	0	16 (20%)
Houts [34], 2010	USA	Mixed/NR	42	55	26	17 (40%)
Krespi Boothby [35], 2010	UK	Breast/Early	255	58	0	22 (9%)
Kugaya [36], 1998	Japan	Mixed/NR	128	61	63	17 (13%)
Lloyd-Williams [37], [38], 2000, 2001	UK	Mixed/Life-expectancy <6 months	100	57	44	22 (22%)
Lloyd-Williams [39], 2007	UK	Mixed/Life-expectancy <6 months	246	62	43	74 (30%)
Love [40], 2002	Australia	Breast/Stages I–IIb (excluding T3, N0, M0)	303	NR	0	29 (10%)
Love [41], 2004	Australia	Breast/Stage IV	227	52	0	16 (7%)
Meyer [42], 2003	UK	Mixed/Terminal	45	NR	42	9 (20%)
Murphy [43], 2006	UK	NR/Advanced metastatic cancer in palliative care	16	68	50	6 (38%)
Özalp [44], 2008	Turkey	Breast/Mixed	204	51	0	17 (8%)
Passik [45], 2001	USA	Mixed/NR	60	58	47	24 (40%)
Patel [46], 2010	Australia	Breast/Mixed (excluding Stage IV)	100	53	0	8 (8%)
Smith [47], 2006	UK	Mixed/NR	381	56	50	40 (10%)
Walker [48], 2007	UK	Mixed/Mixed	361	62	24	30 (8%)

Abbreviations: GAD = General Anxiety Disorder; MDD = Major Depressive Disorder; NR = Not reported; UK = United Kingdom; USA = United States of America.

a Study reported diagnostic accuracy data for all 113 women in the study, and also after excluding women with MDD already treated with antidepressants and women with GAD already treated with antidepressants or anxiolytics (N = 103).

b Mean age based on all 113 women in the study.

c Demographic data are based on full study sample of 109 patients, rather than the 79 patients included in the analyses reported in the table. The authors excluded 30 patients with anxiety or adjustment disorders, but not MDD, from diagnostic accuracy analyses.

**Table 2:** Results of Diagnostic Accuracy of Depression Screening Tools.

First Author, Year	Country	Major Depression Criterion Standard	Instrument/Cutoff	Derivation of Cutoff	Sensitivity % (95% CI)	Specificity % (95% CI)	Positive Predictive Value % (95% CI)	Negative Predictive Value % (95% CI)
Akechi [31], 2006	Japan	SCID	1 item, “Depressed?^a^	NA	79 (52–92)	92 (87–95)	41 (25–59)	98 (95–99)
			1 item, “Lost interest?”^a^	NA	93 (68–99)	92 (87–95)	45 (28–62)	99 (97–100)
			1 item, “Depressed” or “Lost Interest?”^a^	NA	100 (78–100)	86 (81–90)	34 (22–49)	100 (98–100)
			HADS ≥17	Exploratory	71 (45–88)	77 (71–83)	19 (10–31)	97 (93–99)
			HADS-D ≥9	Exploratory	86 (60–96)	69 (62–75)	17 (10–27)	99 (95–100)
Alexander [30], 2010	UK	SCID	EPDS ≥13	Literature	72 (49–88)	90 (85–94)	42 (26–59)	97 (93–99)
			HADS-D ≥11	Literature	50 (29–71)	97 (94–99)	64 (39–84)	95 (91–97)
Coyne [29], 2004	USA	SCID	HSCL-25 ≥44^b^	Literature	70 (40–89)	75 (66–82)	21 (11–38)	96 (90–99)
			HSCL-25 ≥44^b^	Literature	67 (21–94)	74 (65–82)	7 (2–23)	99 (93–100)
Grassi [32], 2009	Italy	CIDI	DT ≥5	Exploratory	79 (52–92)	83 (72–90)	50 (31–69)	95 (86–98)
			HADS ≥15	Exploratory	86 (60–96)	95 (87–98)	80 (55–93)	97 (89–99)
Hopwood [33], 1991	UK	CIS	HADS-D ≥11	Exploratory	75 (51–90)	75 (64–84)	43 (27–61)	92 (82–97)
Houts [34], 2010	USA	SCID	PCM Acute Distress Scale ≥61	Exploratory	100 (82–100)	84 (65–94)	81 (60–92)	100 (85–100)
			PCM Despair Scale ≥63	Exploratory	94 (73–99)	84 (65–94)	80 (58–92)	95 (78–99)
Krespi Boothby [35], 2010	UK	SADS	HADS-D ≥7	Exploratory	77 (57–90)	87 (82–91)	36 (24–50)	98 (95–99)
			GHQ-12 ≥4	Exploratory	77 (57–90)	82 (77–86)	29 (19–41)	97 (94–99)
Kugaya [36], 1998	Japan	SCID	HADS ≥20	Exploratory	82 (59–94)	96 (91–99)	78 (55–91)	97 (92–99)
			HADS-D ≥11	Exploratory	82 (59–94)	96 (90–98)	74 (51–88)	97 (92–99)
			HADS-A ≥8	Exploratory	94 (73–99)	88 (80–92)	53 (36–70)	99 (94–100)
Lloyd-Williams [37]–[38], 2000, 2001	UK	PSE	HADS ≥19	Exploratory	68 (47–84)	67 (56–76)	37 (24–52)	88 (77–94)
			HADS-D ≥11	Exploratory	55 (35–73)	74 (64–83)	38 (23–55)	85 (75–92)
			HADS-A ≥10	Exploratory	59 (39–77)	68 (57–77)	34 (21–50)	85 (75–92)
			EPDS ≥13	Exploratory	82 (61–93)	79 (69–87)	53 (37–69)	94 (85–98)
Lloyd-Williams [39], 2007	UK	PSE	EPDS ≥12	Exploratory	72 (60–81)	74 (67–80)	54 (44–64)	86 (79–91)
			Brief EPDS ≥6	Exploratory	72 (60–81)	83 (77–88)	65 (54–74)	87 (81–91)
Love [40], 2002	Australia	MILP	HADS-D ≥11	Literature	7 (2–22)	98 (95–99)	25 (7–59)	91 (87–94)
Love [41], 2004	Australia	MILP	HADS-D ≥7	Exploratory	81 (57–93)	80 (74–85)	24 (14–36)	98 (95–99)
			BDI-SF ≥5	Exploratory	94 (72–99)	63 (56–69)	16 (10–25)	99 (96–100)
Meyer [42], 2003	UK	SCID	MEQ ≥90	Literature	56 (27–81)	94 (82–98)	71 (36–92)	89 (76–96)
Murphy [43], 2006	UK	SCID	EPDS ≥13	Literature^c^	67 (30–90)	100 (72–100)	100 (51–100)	83 (55–95)
Özalp [44], 2008	Turkey	SCID	HADS ≥17	Exploratory	71 (47–87)	80 (74–85)	24 (15–38)	97 (93–99)
			HADS-D ≥5	Exploratory	88 (66–97)	59 (52–66)	16 (10–25)	98 (94–100)
			HADS-A ≥7	Exploratory	65 (41–83)	69 (62–75)	16 (9–26)	96 (91–98)
Passik [45], 2001	USA	MINI	ZSDS ≥48	Literature/ Exploratory^d^	67 (47–82)	86 (71–94)	76 (55–89)	79 (64–89)
			BZSDS ≥22	Literature/ Exploratory^d^	96 (80–99)	42 (27–58)	52 (38–66)	94 (72–99)
Patel [46], 2010	Australia	CIDI	BC-VI ≥2	Exploratory	88 (53–98)	59 (48–69)	17 (8–30)	98 (90–100)
			HADS-D ≥8	Literature	17 (3–56)	94 (87–98)	20 (4–62)	93 (85–97)
			PSYCH-6 ≥2	Literature	80 (38–96)	68 (56–78)	15 (6–34)	98 (89–100)
Smith [47], 2006	UK	SCAN / PSE	HADS-D ≥7	Exploratory	73 (57–84)	64 (59–69)	19 (14–26)	95 (92–97)
			HADS-D minus misfitting items ≥5	Exploratory	70 (55–82)	60 (55–65)	17 (12–24)	94 (91–97)
Walker [48], 2007	UK	SCID	HADS ≥15	Exploratory	87 (70–95)	85 (81–88)	34 (25–45)	99 (96–99)
			HADS-D ≥7	Exploratory	90 (74–97)	88 (84–91)	40 (29–52)	99 (97–100)
			HADS-A ≥9	Exploratory	87 (70–95)	83 (79–87)	32 (23–42)	99 (96–99)

Abbreviations: BC-VI = Breast Cancer - Vulnerability Index; BDI-SF = Beck Depression Inventory Short Form; BZSDS = Brief Zung Self Rating Depression Scale; CIDI = Composite International Diagnostic Interview; CIS = Clinical Interview Schedule; DT = Distress Thermometer; EPDS = Edinburgh Postnatal Depression Scale; GHQ = General Health Questionnaire; HADS = Hospital Anxiety and Depression Scale total score; HADS-A = Anxiety subscale of Hospital Anxiety and Depression Scale; HADS-D = Depression subscale of Hospital Anxiety and Depression Scale; HSCL-25 = 25-item version of the Hopkins Symptom Checklist; MILP = Monash Interview for Liaison Psychiatry; MINI = Mini-International Neuropsychiatric Interview; NA = Not applicable; PCM = Patient Care Monitor; PSE = Present State Examination; PSYCH-6 = 6-item subscale measuring symptoms of depression and anxiety from the Somatic and Psychological Health Report (SPHERE); SADS: Schedule for Affective Disorders and Schizophrenia; SCAN = Schedule for Clinical Assessment in Neuropsychiatry; SCID = Structured Clinical Interview for DSM; ZSDS = Zung Self Rating Depression Scale.

a Items were embedded in the diagnostic interview, and at least 1 of 2 was required for a diagnosis of major depression.

b Study reported diagnostic accuracy data for all 113 women in the study (first line), and also after excluding women with MDD already treated with antidepressants and women with GAD already treated with antidepressants or anxiolytics (N = 103; second line).

c A cutoff of 13 or greater on the EPDS is standard, although the authors did not indicate this explicitly.

d Authors used several different cutoffs from the literature and tested to determine optimal cutoff in their sample.

There were 6 studies [31], [32], [36]–[38], [44], [48] of the Hospital Anxiety and Depression Scale (HADS). The 6 studies included between 14 and 30 MDD cases. All used exploratory ROC methods, and they identified optimal screening cutoffs that ranged from 15 to 20. Nine studies [31], [33], [35]–[38], [41], [44], [47], [48] with 14 to 40 MDD cases per study, used ROC methods with the HADS depression subscale (HADS-D) and reported optimal cutoff scores from 5 to 11. Only 3 studies [30], [40], [46] used *a priori* defined standard cutoffs, 8 [46] or 11 [30], [40], to assess diagnostic accuracy with the HADS-D and reported sensitivities of 7% to 50%. Two studies [37]–[39] used ROC methods with the Edinburgh Postnatal Depression Scale (EPDS) and identified optimal cutoff scores of 12 and 13, similar to the standard cutoff of 13 used in two other studies [30], [43]. Excluding a study with only 6 MDD cases [43], sensitivity with the EPDS ranged from 72% to 82%, specificity from 74% to 90%, positive predictive value from 42% to 54%, and negative predictive value from 86% to 97%. Apart from the HADS anxiety subscale, no other screening tool was used in more than one study (see Table 2). One study [29] assessed the yield of screening with and without excluding patients with psychiatric disorders already treated with psychotropic medications and found that the true positive rate of depression screens fell from 21% to 7% after excluding patients who were already receiving treatment prior to screening.

As shown in Table 3, the methodological quality of the 19 diagnostic accuracy studies was generally adequate for administering the same reference test to all patients in the study; for the reference being independent of the screening test; and for adequately describing the screening and diagnostic tests. However, 17 of 19 studies failed to exclude patients who were already diagnosed or receiving depression treatment and who would not be newly identified through screening. In addition, 6 studies were rated ‘no’ or ‘unclear’ for clear sample selection criteria, 10 for timing of the screening tool and diagnostic interview administration, 11 for blind interpretation of the diagnostic interview, 19 for description of handling of missing data, and 8 for explanation of study withdrawals.

**Table 3:** Quality Assessment of Studies of Diagnostic Accuracy (QUADAS).

	QUADAS Items^a^										
First Author, Year	#1 Patient Spectrum^b^	#2 Selection Criteria Clear	#4 Timing of Ref and Index Tests^c^	#5 Whole Sample Received Ref Test	#6 All Patients with Same Ref Test	#7 Ref Indep of Index Test	#8 Index Test Descrip	#9 Ref Test Descrip	#11 Ref Interpret Blind to Index	#13 Missing Data^d^	#14 Study With-drawals
Akechi [31], 2006	No	Yes	Yes	Yes	Yes	Yes (HADS) No (single item)	Yes	Yes	Unclear (HADS) No (single item)	Unclear	No
Alexander [30], 2010	No	Yes	Unclear	Yes	Yes	Yes	Yes	Yes	Unclear	Unclear	Yes
Coyne [29], 2004	Yes	Yes	Unclear	Yes	Yes	Yes	Yes	Yes	Unclear	Unclear	Unclear
Grassi [32], 2009	No	Yes	Yes	Yes	Yes	Yes	Yes	Yes	Yes	Unclear	No
Hopwood [33], 1991	No	Yes	Unclear	No	Yes	Yes	Yes	No	Yes	Unclear	Yes
Houts [34], 2010	No	No	Yes^e^	Yes	Yes	Yes	Yes	Yes	Yes	Unclear	Yes
Krespi Boothby [35], 2010	No	Unclear	Yes	Yes	Yes	Yes	Yes	No	Yes	Unclear	No
Kugaya [36], 1998	No	No	Yes	Yes	Yes	Yes	Yes	Yes	Yes	Unclear	No
Lloyd-Williams [37], [38], 2000, 2001	Yes	Yes	Unclear	Yes	Yes	Yes	Yes	Yes	Yes	Unclear	Yes
Lloyd-Williams [39], 2007	No	Yes	Unclear	Yes	Yes	Yes	Yes	Yes	Yes	Unclear	Yes
Love [40], 2002,	No	Yes	Unclear	Yes	Yes	Yes	Yes	Yes	Unclear	Unclear	Unclear
Love [41], 2004	No	Yes	Unclear	Yes	Yes	Yes	Yes	Yes	Unclear	Unclear	Unclear
Meyer [42], 2003	No	Yes	Yes	Yes	Yes	Yes	Yes	Yes	Unclear	Unclear	Yes
Murphy [43], 2006	No	Unclear	Yes	Yes	Yes	Yes	Yes	Yes	Unclear	Unclear	Yes
Özalp [44], 2008	No	Yes	Yes	Yes	Yes	Yes	Yes	Yes	Yes	Unclear	Yes
Passik [45], 2001	No	No	Unclear	Yes	Yes	Yes	Yes	Yes	No	Unclear	Yes
Patel [46], 2010	No	Yes	No^f^	Yes	Yes	Yes	Unclear	Yes	Unclear	Unclear	Yes
Smith [47], 2006	No	No	Yes	Unclear	Yes	Yes	Yes	Yes	Unclear	Unclear	Unclear
Walker [48], 2007	No	Yes	No	Yes	Yes	Yes	Yes	Yes	Unclear	Unclear	Yes

a See Supplementary Information S5 for QUADAS items. Items are rated ‘yes’, ‘no’ and ‘unclear’ based on the user's guide^23^ and reflecting the likelihood of being free of bias. Items #3 (reference standard appropriate), #10 (blind interpretation of test results) and #12 (same clinical data available as in practice) were not evaluated because an appropriate reference standard was a criterion for review eligibility and because scoring of all self-report depression screening tools is fully automated and does not require judgment.

b Item #1 scored ‘no’ if patients with already diagnosed or treated depression were not excluded from study sample as they would not constitute newly identified cases in clinical practice. Studies were not downgraded for only sampling one type or stage of cancer.

c Item #4 scored ‘yes’ if index test and reference standard were administered within 1 week of each other, ‘no’ if longer, and ‘unclear’ if not specified. Studies in which a significant number of patients received assessments more than 2 weeks apart were not included in the systematic review.

d Item #13 originally was “Were uninterpretable, indeterminate or intermediate test results reported?” This item was adapted as “Were missing data on the index test handled correctly?”.

e Authors clarified that most patients received the index test and reference standard on the same day and all within 5 days.

f Authors clarified that 67% of interviews were conducted within one week and 93% within 2 weeks.

### Key Question #2: Effect of Depression Treatment

**Figure 3:**
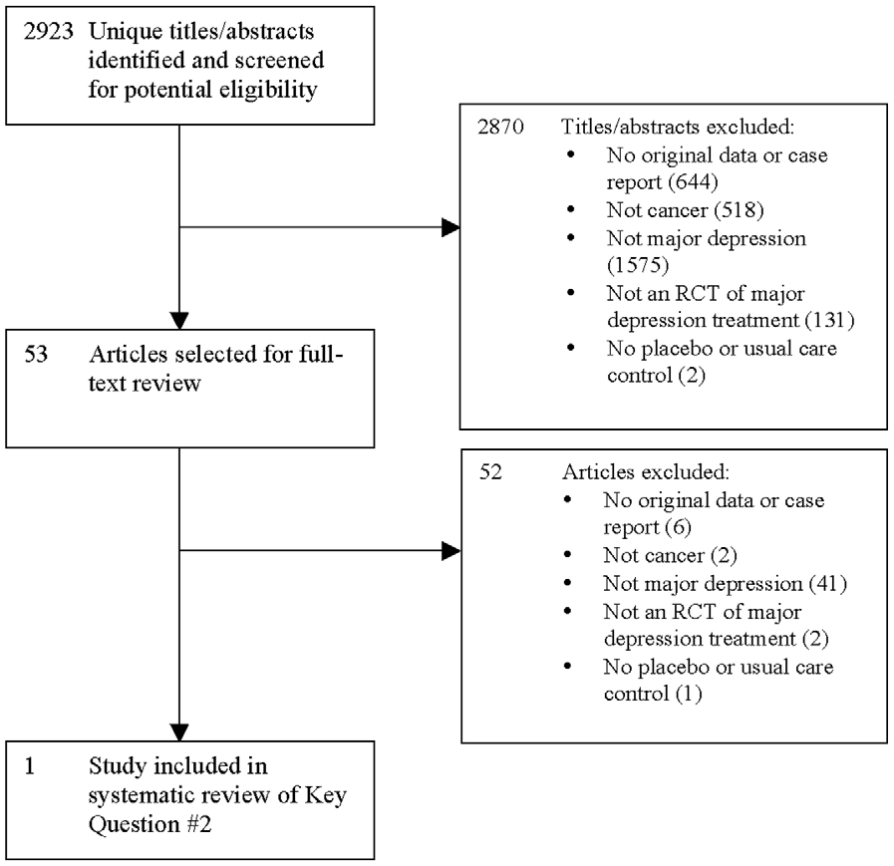
PRISMA Flow Diagram of Study Selection Process for Key Question #2.

For Key Question #2, 2,923 unique citations were identified. As shown in Figure 3, 2,870 were excluded after title/abstract review, and 52 after full-text review, leaving 1 eligible RCT. That study [49] of patients with MDD based on the SCID-IV randomized 99 patients to usual cancer care and 101 to usual care plus a nurse-delivered collaborative care depression intervention (Table 4). The intervention involved up to 10 one-to-one sessions (mean = 7) over 3 months. Sessions included education about depression and its treatment, problem-solving and coping strategies, and communication with physicians about depression management. Study nurses reviewed each patient's progress with a psychiatrist weekly and communicated with the patient's primary care physician regarding patient progress and psychiatrist recommendations. Post-intervention depression scores were significantly reduced compared to the usual care group (Hedges's *g* = 0.37) (see Table 5). Study quality was high (Table 6).

**Table 4:** Characteristics of Randomized Controlled Trial of Depression Treatment.

First Author,Year	Study Funding Source	Cancer Site/Description	Treatment vs. Control	Number of Patients Randomized	Mean Age (Years)	Males (%)
Strong [49], 2008	Non-Industry	Mixed/Mixed	Nurse Intervention vs. UC	Total: 200; Tx: 101; UC: 99	Total: 57; Tx: 57; UC: 57	Total: 30%; Tx: 31%; UC: 28%

Abbreviations: Tx = treatment; UC = usual care.

**Table 5:** Results of Randomized Controlled Trial of Depression Treatment.

First Author,Year	Number (%) Lost to Follow-up	Treatment Duration	Depression Outcomes^a^			
			Remission:^b^ N (%) and Relative Risk Ratio (95% CI)	Response:^c^ N (%) and Relative Risk Ratio (95% CI)	Primary Outcome: Hedges's *g* (95% CI) and r^2^	Secondary Outcome(s): Hedges's *g*(95% CI) and r^2^
Strong [49], 2008	Total: 4 (2%); Tx: 0 (0%); UC: 4 (4%)	Mean of 7 sessions over 3 months	(a) Tx^d^ 28 (28%); UC: 14 (14%); RR = 2.0 (1.1 to 3.5); (b) Tx^e^ 65 (64%); UC: 44 (44%); RR = 1.4 (1.1 to 1.9)	(a) Tx^d^ 51 (50%); UC: 34 (34%); RR = 1.5 (1.1 to 2.1)	SCL-20 depression f *g* = 0.37 (0.09 to 0.65); r^2^ = 0.03	NR

Abbreviations: CI = confidence interval; NR = not reported; RR = relative risk ratio; SCL-20 depression = depression subscale derived from the Symptom Checklist-90; Tx = treatment; UC = usual care.

a Depression outcomes were assessed at the end of the treatment period. Continuous outcomes that favored the treatment group are reported in this table as positive numbers.

b Remission defined as (a) <0.75 on the SCL-20 and (b) no longer having major depression based on the SCID-IV.

c Response defined as a 50% reduction in SCL-20 score from baseline.

d Publication included remission and response data for 97 patients in the intervention group and 99 in the usual care group. In this table, patients lost to follow-up are counted as non-remitters and non-responders.

e Publication included remission data for 96 patients in the intervention group and 98 in the usual care group. In this table, patients lost to follow-up are counted as non-remitters.

f Unadjusted effect size *g* calculated from mean SCL-20 scores 3 months post-randomization for 97 patients randomized to the intervention group and 99 randomized to the usual care group, as shown in Table 2 of Strong et al.^50^

**Table 6:** Assessment of Risk of Bias in Randomized Controlled Trial in Key Question #2 (Treatment).

	Cochrane Risk of Bias Tool Items^a^						
First Author, Year	#1 Sequence Generation	#2 Allocation Concealment	#3 Blinding	#4 Incomplete Outcome Data	#5 Selective Outcome Reporting	#6 Other Sources of Bias	#7 Overall Risk of Bias Rating
Strong [49], 2008	low	low	uncertain	low	low	low	low

a See Supplementary Information S6 for item descriptions. Items are scored as ‘high’, ‘low’, or ‘uncertain’ risk of bias.

### Key Question #3: Effect of Depression Screening

**Figure 4:**
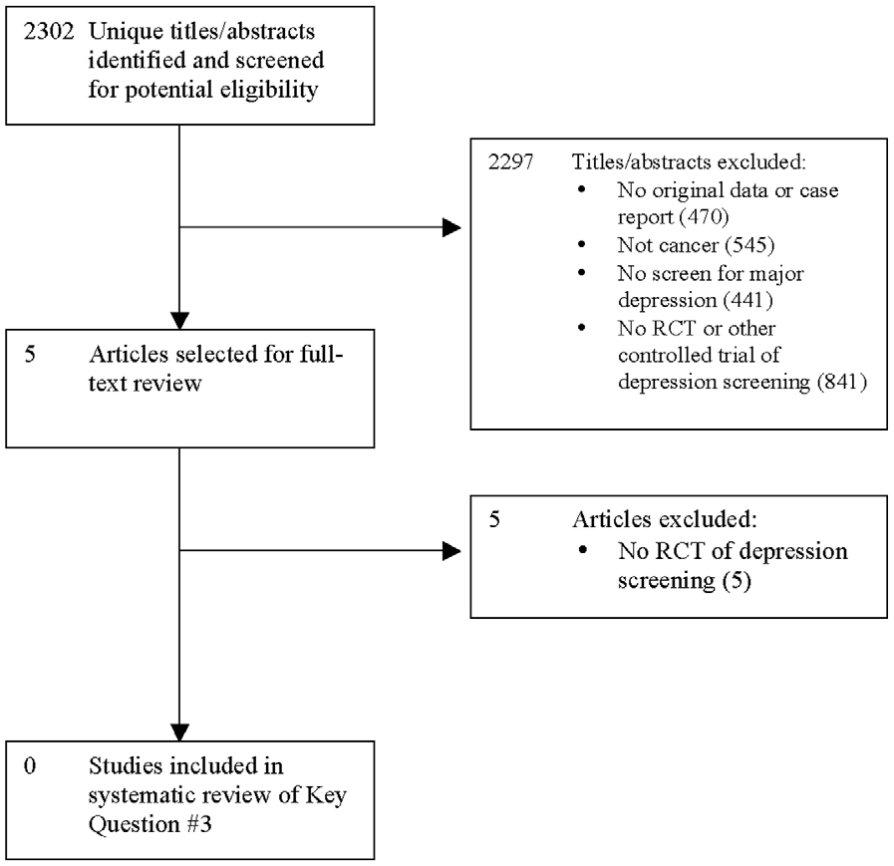
PRISMA Flow Diagram of Study Selection Process for Key Question #3.

Of 2,302 unique titles/abstracts from the database search, 5 were selected for full-text review, and no RCTs of depression screening met review eligibility criteria (Figure 4).

A number of other studies (see Table S1) described by their authors or in other reviews as related to screening were excluded from the present systematic review. Several were excluded because they did not use a positive depression screen based on a pre-specified cutoff score to determine which patients would receive further assessment or treatment. In those studies, a range of screening tools was often made available for clinical consultations, but scores on a depression screening tool did not determine referral for psychosocial evaluation or treatment. Studies were also excluded because they (1) were not RCTs; (2) included multiple screening tools for many different problems, not allowing the effect of depression screening to be evaluated separately; or (3) did not report depression symptom or diagnosis outcomes.

## Discussion

One of the most important functions of systematic reviews is to identify areas where there is not sufficient evidence and where clinical trials are needed [50]. The main finding of this systematic review was that there are no RCTs that have evaluated whether screening for depression among cancer patients would improve depression outcomes. This is important because reports from an NIH panel [8] and the IOM [4] and clinical guidelines from the NCCN [3] and NICE [7] have recommended that screening for psychological distress, including depression, be part of standard supportive and palliative cancer care. The results of this systematic review show that these recommendation statements are not supported by evidence from RCTs that screening cancer patients for depression would improve patients' mental health beyond existing psychosocial services that are offered in oncology settings.

As described in well-established criteria for evaluating the potential benefit of screening programs [10], [12] and methods developed by the USPSTF [14] in the absence of evidence from well-conducted RCTs on the benefits versus harms of screening it is important to examine whether evidence on the performance of screening tools and the efficacy of treatment is sufficiently robust as to warrant recommendations for screening and where there are gaps in the process that require more research.

With respect to the accuracy of depression screening tools in cancer settings, most studies that we reviewed used exploratory methods that identify cutoff scores that maximize diagnostic accuracy in a particular sample. These methods tend to yield inflated estimates of screening accuracy that do not replicate consistently in other samples [51]. In addition, sample sizes were generally small for the purpose of assessing diagnostic accuracy with a median of 17 MDD cases per study. Not surprisingly, optimal cutoff scores for the two instruments that were used most frequently, the HADS and HADS-D, varied too widely to provide guidance to clinicians on their optimal use. Optimal cutoffs ranged from 15 to 20 for the HADS and 5 to 11 for the HADS-D. Three studies that used *a priori* defined standard cutoffs for the HADS-D reported very low sensitivity (7% to 50%). The accuracy of the EPDS was better, with cutoffs of 12 and 13 producing reasonably high sensitivity (72–82%) and specificity (74–90%) estimates, although only one study included more than 22 patients with MDD. All studies for Key Question #1 were based on samples that included already diagnosed and treated patients. This would be expected to generate inflated estimates of screening sensitivity and exaggerate the number of previously undetected cases that would be identified through screening in clinical practice as described in a recent overview [52].

With respect to depression treatment, we identified 1 high-quality RCT of a nurse-delivered collaborative care intervention for MDD [49]. That study found that cancer patients randomized to the intervention experienced a small to moderate reduction in depressive symptoms (Hedges's *g* = 0.37), similar to the estimated effect reported in a meta-analysis of collaborative care interventions in primary care (standardized mean effect size = 0.25) [53]. A number of studies have used psychosocial interventions to address a range of clinical domains associated with cancer, but not MDD, and were not included in this review [54]. A collaborative care intervention [55] and several antidepressant trials for depression [54] were also excluded because they defined MDD based on non-validated clinician interviews or scores on self-report questionnaires. Results from those studies generally support the conclusion that depression treatment is similarly effective for patients with and without cancer [54], [55].

The nurse-delivered collaborative care intervention trial reported by Strong et al. [49] tested the kind of integrated depression care that might be considered for patients identified as depressed in a screening program. This trial was included in the review of treatment effects, but not the effects of screening, because it only enrolled patients who had been diagnosed with MDD. Thus, the results of the trial suggest that collaborative care would improve outcomes for patients already identified as depressed. They do not, however, address the important question of whether patients from a cancer setting who are screened would have better outcomes than patients who are not screened, but who could receive collaborative depression care after referral by a healthcare provider outside of the context of screening. Per standard criteria for evaluating screening programs [10]–[12], RCTs of screening assess outcomes for patients screened versus patients not screened. Thus, an important limitation of our review was that there were no RCTs that compared depression outcomes among patients screened for depression compared to patients not screened for depression.

### Depression Screening in Context

Depression screening is only useful to the degree that it leads to improved outcomes above and beyond existing care. Thus, to be successful, a screening program would need to identify a meaningful number of patients as depressed out of those who have opted not to utilize available psychosocial supports; successfully enroll those patients in treatment; and achieve positive treatment results. As illustrated by one study from Germany [56], however, the desire for psychosocial support to cope with cancer may not be correlated with distress levels, and nearly as many patients with low levels of distress may desire supportive care as patients above the cutoff criterion on a screening tool. To provide incremental benefit to patients, depression screening programs in cancer must be able to uncover and address unmet needs [57].

As described in the recently updated NICE guidelines for depression care in general medical settings, it should not be assumed that screening programs would necessarily meet currently unmet care needs. The NICE guidelines noted a lack of evidence for benefit from depression screening and, therefore, rather than routine screening of all patients, recommended strategies to identify depression among high-risk groups of patients or patients otherwise identified by physicians as possibly having depression [58]. In addition to the overall lack of evidence for benefits from screening, the authors of the NICE report cited a number of other important considerations, including the relatively small proportion of patients who screen positive on screening tools who actually have depression. They noted that many patients who screen positive are mildly depressed and are likely to recover without formal intervention, and that ineffective screening could divert scarce resources from more seriously depressed patients who may receive inadequate treatment as a result [58], [59].

Based on existing evidence from other patient groups, it is clear that screening without comprehensive systems for depression assessment and management does not improve depression outcomes. There are at least 11 trials in primary care [60], for instance, that have tested whether screening and referral for depression treatment improves depression outcomes, and all have been negative. Some of these primary care trials have found that screening increases the number of patients treated for depression, but increasing treatment without symptom reduction would be costly and could expose patients to unnecessary harms from treatment without benefit [60]. Thus, the USPSTF recommends depression screening in primary care only when supported by integrated, staff-assisted depression management programs [61]. However, it is not clear whether screening in the context of staff-assisted, collaborative care depression management programs would benefit patients [62], and it is important to differentiate between the effectiveness of screening and the effectiveness of collaborative care. The results of the collaborative care treatment trials reviewed by the USPSTF suggest that providing collaborative depression care is better than not providing this care. They do not, however, demonstrate that patients who receive screening will have better depression outcomes compared to patients who are not screened when the same treatment and care resources are made available to both groups [9]. This is because, as in the Strong et al. study [49], in the studies reviewed by the USPSTF, patients were required to have depressive symptoms or a diagnosis of depression to be eligible for the trial. In addition, only patients with depression in the intervention groups received a collaborative care intervention for depression, whereas depressed patients in the control groups received only standard care. In actual clinical settings, patients receive the optimal treatment available, whether they are identified through a screening program or via physician recognition. Thus, these trials do not address the issue of whether screening would benefit patients with previously unrecognized depression. Underlining this issue, in the largest of the trials cited by the USPSTF a substantial portion of patients were already recognized and being treated for depression prior to enrolling in the trial and receiving augmented care [9].

### Potential Harms from Depression Screening in Cancer Care

In the absence of demonstrated benefit, potential harms from depression screening for cancer patients should be considered carefully, as outlined in standard evaluative frameworks [10]–[12] and in the USPSTF methodology [14]. The degree to which routine depression screening of patients with cancer might lead to inappropriate labeling and treatment on the one hand, or to extraordinary and impractical overuse of important health care resources, on the other, has not been examined. Routine depression screening would increase the number of cancer patients diagnosed with depression and treated with antidepressant drugs [29], [63]. As a consequence, more patients with cancer would be exposed to potentially harmful drug-drug interactions between antidepressants and either cancer chemotherapeutic agents [63]–[67] or anti-emetics [68]. Interactions between anti-cancer drugs and antidepressants are of particular concern because small alterations in the plasma concentrations of certain members of either drug class can lead to either subtherapeutic effects or drug toxicity [64]. Perhaps of greatest importance is the potential interaction between certain antidepressants and tamoxifen, commonly used as adjuvant therapy for women with breast cancer. The hepatic enzyme CYP2D6 is the principal enzyme that converts tamoxifen to its active metabolite, endoxifen [67]. Some antidepressants, particularly paroxetine, fluoxetine, and bupropion, are strong inhibitors of CYP2D6 and may diminish the therapeutic effect of tamoxifen [29], [65], [66]. Indeed, one study estimated that there would be 1 additional breast cancer death within 5 years of stopping adjuvant treatment for every 20 women who used paroxetine approximately 40% of the time they took tamoxifen [63].

### Conclusions

In summary, this systematic review did not identify any RCTs that compared the benefits versus harms of depression screening in patients with cancer. In the absence of such RCTs, there currently is not evidence to support recommendations for the incorporation of routine depression screening into standard cancer care. Depression treatment appears to be as effective in cancer care as in other settings, but important limitations in the evidence base on screening tools in this population were identified, and research is needed to address these limitations. In order to inform health care providers who must decide whether or not to screen cancer patients for depression and developers of guidelines for cancer care, well-designed and executed RCTs that investigate depression screening programs are needed. Specifically, screening for depression in a cancer treatment setting should be tested in a trial where all patients identified as depressed via screening or by physician recognition and referral in a control group have access to high-quality, integrated depression care. Given the current absence of evidence on the effectiveness of screening in cancer, and the absence of positive results from any trial in other patient groups, however, recommendations for depression screening among patients with cancer are at this point premature.

## Supporting Information

Supplementary Information S1 **Search Strategies for Key Questions #1 and #3 (through January 24, 2011).**
(DOC)

Supplementary Information S2 **Relevant Systematic Reviews.**
(DOC)

Supplementary Information S3 **Journals Included in Manual Searching.**
(DOC)

Supplementary Information S4 **Variables Included in Data Extraction Form.**
(DOC)

Supplementary Information S5 **Quality Assessment of Diagnostic Accuracy Studies (QUADAS) - items scored yes, no, or unclear** ^26^ **.**
(DOC)

Supplementary Information S6 **The Cochrane Tool for Assessing Risk of Bias** ^80^ **.**
(DOC)

Table S1 **Excluded Studies for Effect of Screening on Depression Outcomes (Key Question #3).**
(DOCX)
