# Supplementary material for: Depression Screening and Patient Outcomes in Cancer: A Systematic Review
Source: PLoS One. 2011 Nov 14;6(11):e27181. doi: 10.1371/journal.pone.0027181 (PMC3215716; doi:10.1371/journal.pone.0027181)
Supplement: Supplementary Information S1 — Search Strategies for Key Questions #1 and #3 (through January 24, 2011). (DOC) [file pone.0027181.s001.doc]

**Supplementary Information 1: Search Strategies for Key Questions #1 and #3 (through January 24, 2011)**

**MEDLINE (from 1950):**

(Depression [MeSH] OR “depressive disorder” [MeSH] OR “major depressive disorder” [MeSH] OR “depress*” [tiab] OR distress [tiab]) AND (screen* [tiab] OR “mass screening” [MeSH] OR detect* [tiab] OR assess* [tiab] OR thermometer [tiab] OR interview [tiab] OR instrument [tiab] OR questionnaires [MeSH] OR questionnaire* [tiab] OR inventor* [tiab] OR predict* [tiab] OR scale* [tiab] OR evaluat* [tiab] OR prevalence [MeSH]) AND (“sensitivity and specificity” [MeSH] OR sensitivit* [tiab] OR specificit* [tiab] OR validat* [tiab] OR accura* [tiab]) AND (cancer [MeSH] OR cancer [tiab] neoplasms [MeSH] OR malignanc* [tiab] OR tumor [tiab] OR tumour [tiab])

**Cochrane:**

#1 MeSH descriptor depressive disorder explode all trees

#2 MeSH descriptor depression

#3 depress*: ti,ab,kw

#4 distress: ti,ab,kw

#5 (#1 OR #2 OR #3 OR #4)

#6 MeSH descriptor sensitivity and specificity explode all trees

#7 MeSH descriptor predictive value of tests explode all trees

#8 sensitiv* : ti,ab,kw

#9 specific*: ti,ab,kw

#10 validat*: ti,ab,kw

#11 accura*: ti,ab,kw

#12 (#6 OR #7 OR #8 OR #9 OR #10 OR #11)

#13 MeSH descriptor psychological tests explode all trees

#14 MeSH descriptor psychiatric status rating scales explode all trees

#15 MeSH descriptor mass screening explode all trees

#16 MeSH descriptor interview, psychological explode all trees

#17 MeSH descriptor prevalence explode all trees

#18 MeSH descriptor self assessment (psychology) explode all trees

#19 MeSH descriptor questionnaires explode all trees

#20 predict*: ti,ab,kw

#21 detect*: ti,ab,kw

#22 assess*: ti,ab,kw

#23 evaluat*: ti,ab,kw

#24 interview: ti,ab,kw

#25 instrument*: ti,ab,kw

#26 scale*: ti,ab,kw

#27 screen*: ti,ab,kw

#28 score: ti,ab,kw

#29 questionnaire*: ti,ab,kw

#30 inventor*: ti,ab,kw

#31 evaluat*: ti,ab,kw

#32 thermometer: ti,ab,kw

#33 (#13 OR #14 OR #15 OR #16 OR #17 OR #18 OR #19 OR #20 OR #21 OR #22 OR #23 OR #24 OR #25 OR #26 OR #27 OR #28 OR #29 OR #30 OR #31 OR #32)

#34 MeSH descriptor neoplasms explode all trees

#35 cancer: ti,ab,kw

#36 neoplasm*: ti,ab,kw

#37 malignan*: ti,ab,kw

#38 tumor: ti,ab,kw

#39 tumour: ti,ab,kw

#40 (#34 OR #35 OR #36 OR #37 OR #38 OR #39)

#41 (#5 AND #12 AND #33 AND #40)

**PsycINFO (from 1887):**

S1: 1. “major depression” MJ

OR 2. “depression (emotion)” MJ

OR 3. Depress* TI

OR 4. Distress MJ

OR 5. Distress TI

S2: 1. “screening tests” MJ

OR 2. “psychological screening inventory” MJ

OR 3. screen* TI

OR 4. screen* AB

OR 5. detect* TI

OR 6. assess* TI

OR 7. Thermometer TI

OR 8. interview TI

OR 9. Instrument TI

OR 10. questionnaire* TI

OR 11. inventor* TI

OR 12. predict* TI

OR 13. scale* TI

OR 14. evaluat* TI

OR 15. Prevalence TI

S3: 1. sensitiv* TI

OR 2. Sensitiv* AB

OR 3. specific* TI

OR 4. Specific* AB

OR 5. validat* TI

OR 6. accura* TI

S4: 1. Neoplasms MJ

OR 2. Cancer TI

OR 3. Cancer AB

OR 4. malignan* TI

OR 5. Malignan* AB

OR 6. Tumor TI

OR 7. Tumor AB

OR 8. Tumour TI

OR 9. Tumour AB

S5: S1 AND S2 AND S3 AND S4

**CINAHL (from 1981):**

S1: 1. Depress* TI

OR 2. Depress* AB

OR 3. distress TI

OR 4. Distress AB

S2: 1. psychological tests MJ

OR 2. questionnaires MJ

OR 3. Screen* TI

OR 5. Detect* TI

OR 6. Assess* TI

OR 7. Predict* TI

OR 8. Thermometer TI

OR 10. Interview TI

OR 11. Instrument TI

OR 12. Evaluat* TI

OR 13. Inventor* TI

OR 14. Scale* TI

OR 15.  "sensitivity and specificity" MJ

OR 16. "reliability and validity" MJ

OR 17. sensitiv* TI

OR 18. sensitiv* AB

OR 19. specific* TI

OR 20. Specific* AB

OR 21. validat* TI

OR 22. accura* TI

S3 1. Neoplasms MJ

OR 2. cancer TI

OR 3. Cancer AB

OR 4. malignan* TI

OR 5. Tumor TI

OR 6. Tumour TI

S4 S1 AND S2 AND S3

**EMBASE (from 1974):**

1. depression/mj OR depress*:ti,ab OR “distress syndrome”/mj OR distress:ti,ab

2. screening/mj OR screen*:ti,ab OR detect*:ti,ab OR assess*:ti,ab OR thermometer:ti,ab OR “psychologic test”/mj OR interview/mj OR instrument:ti,ab OR questionnaire/mj OR inventor*:ti,ab OR predict*:ti,ab OR scale*:ti,ab OR evaluat*:ti,ab OR prevalence:ti,ab

3. “sensitivity and specificity”/mj OR sensitivit*:ti,ab OR specificit*:ti,ab OR validat*:ti,ab OR accura*:ti,ab

4. neoplasm/mj OR cancer:ti,ab OR malignan*:ti,ab OR tumor:ti,ab OR tumour:ti,ab

5. 1 and 2 and 3 and 4

* Map to preferred terminology, include sub-terms/derivatives (explosion search), humans

**ISI (from 1900):**

1. TS=(major depressive disorder) OR TS=depression OR TI=depress* OR TS=distress OR TI=distress

2. TS=screening OR TI=screen* OR TI=detect* OR TI=assess* OR TI=thermometer OR TI=interview OR TI=instrument OR TI=questionnaire* OR TI=inventor* OR TI=predict* OR TI=scale* OR TI=evaluat*

3. TS=sensitivity OR TI=sensitiv* OR TS=specificity OR TI=specific* OR TI=valid* OR TI=accura*

4. TS=neoplasms OR TI=neoplasm* OR TI=malignan* OR TI=cancer OR TI=tumor OR TI=tumour

5. #1 AND #2 AND #3 AND #4

* Advanced search, all years, no restrictions

**SCOPUS (from 1869):**

TITLE-ABS-KEY (“major depressive disorder” OR depress* OR distress) AND TITLE (screen* OR detect* OR assess* OR thermometer OR interview OR instrument OR questionnaire* OR inventor* OR predict* OR scale* OR evaluat* OR prevalence OR sensitiv* OR specific* OR validat* OR accura*) AND TITLE-ABS-KEY (neoplasm* OR cancer OR malignan* OR tumor OR tumour)

**Search Strategies for Key Question #2 (through January 24, 2011)**

**MEDLINE (from 1950):**

(Depression [MeSH] OR “depressive disorder” [MeSH] OR “major depressive disorder” [MeSH] OR “depress*” [tiab] OR distress [tiab]) AND (Therapeutics [MeSH] OR therapy [tiab] OR “drug therapy” [MeSH] OR intervention [tiab] OR pharmacological [tiab] OR “antidepressive agents” [MeSH] OR antidepress* [tiab] OR SSRI [tiab] OR psychotherapy [MeSH] OR treatment [tiab] OR psychologic [tiab] OR “cognitive therapy” [MeSH] OR “behavior therapy” [MeSH] OR “treatment outcome” [MeSH]) AND (cancer [MeSH] OR neoplasms [MeSH] OR malignancy [tiab] OR tumor [tiab] OR tumour [tiab])

* Humans, clinical trial, randomized controlled trial

**Cochrane:**

#1 MeSH descriptor depressive disorder explode all trees

#2 MeSH descriptor depression

#3 depress*: ti,ab,kw

#4 distress: ti,ab,kw

#5 (#1 OR #2 OR #3 OR #4)

#6 MeSH descriptor therapeutics explode all trees

#7 MeSH descriptor psychotherapy explode all trees

#8 MeSH descriptor treatment outcome explode all trees

#9 MeSH descriptor antidepressive agents explode all trees

#10 therapy: ti,ab,kw

#11 intervention: ti,ab,kw

#12 pharmacological: ti,ab,kw

#13 antidepress*: ti,ab,kw

#14 SSRI: ti,ab,kw

#15 treatment: ti,ab,kw

#16 psychotherapy: ti,ab,kw

#17 psychological: ti,ab,kw

#18 (#6 OR #7 OR #8 OR #9 OR # 10 OR #11 OR #12 OR #13 OR #14 OR #15 OR #16 OR #17)

#19 MeSH descriptor neoplasms explode all trees

#20 cancer: ti,ab,kw

#21 neoplasm*: ti,ab,kw

#22 malignanc*: ti,ab,kw

#23 tumor: ti,ab,kw

#24 tumour: ti,ab,kw

#25 (#19 OR # 20 OR #21 OR #22 OR #23 OR #24)

#26 (randomized AND controlled AND trial): publ.type

#27 (#5 AND #18 AND #25 AND #26)

**PsycINFO (from 1887):**

S1: 1. “major depression” MJ

OR 2. “depression (emotion)” MJ

OR 3. Depress* TI

OR 4. Distress MJ

OR 5. Distress TI

S2: 1. Treatment MJ

OR 2. Therapy TI

OR 3. Therapy AB

OR 4. Intervention TI

OR 5. Intervention AB

OR 6. Pharmacologic* TI

OR 7. Pharmacologic* AB

OR 8. antidepress* TI

OR 9. antidepress* AB

OR 11. Psychotherapy TI

OR 12. psychotherapy AB

S3: 1. Neoplasms MJ

OR 2. Cancer TI

OR 3. malignan* TI

OR 4. Tumor TI

OR 5. Tumor AB

OR 6. Tumour TI

OR 7. Tumour AB

S4: S1 AND S2 AND S3

**CINAHL (from 1981):**

S1: 1. Depress* TI

OR 2. Depress* AB

OR 3. distress TI

OR 4. Distress AB

S2: 1. "drug therapy" MJ

OR 2. psychotherapy MJ

OR 3. Treatment TI

OR 4. therapy TI

OR 5. Intervention TI

S3: 1. Neoplasms MJ

OR 2. cancer TI

OR 3. Cancer AB

OR 4. malignan* TI

OR 7. Tumor TI

OR 9. Tumour TI

S4: S1 AND S2 AND S3

* Limit to clinical trial, humans

**EMBASE (from 1974):**

1. depression/mj OR depress*:ti,ab OR “distress syndrome”/mj OR distress:ti,ab

2. therapy/mj OR “intervention study”/mj OR pharmacological:ti,ab OR “antidepressant agent”/mj OR antidepress*:ti,ab OR SSRI:ti,ab OR psychotherapy/mj OR treatment:ti,ab

3. neoplasm/mj OR cancer:ti,ab OR malignan*:ti,ab OR tumour:ti,ab

4. 1 AND 2 AND 3

* Map to preferred terminology, include sub-terms/derivatives (explosion search), humans, controlled clinical trial, randomized controlled trial

**ISI (from 1900):**

1. TS=(major depressive disorder) OR TS=depression OR TI=depress* OR TS=distress OR TI=distress

2. TS=Therapeutics OR TS=therapy OR TI=drug therapy OR TI=intervention OR TI=treatment OR TI=pharmacological OR TI=psychological OR TI=antidepress* OR TI=psychotherapy OR TI=cognitive OR TI=behavior OR TI=outcome OR TI=effect*

3. AND TS=controlled

4. TS=neoplasms OR TI=neoplasm* OR TI=malignan* OR TI=cancer OR TI=tumor OR TI=tumour

5. #1 AND #2 AND #3 AND #4

**SCOPUS (from 1869):**

(TITLE (“major depressive disorder”) OR TITLE (depress*) OR TITLE (distress)) AND (TITLE (therapeutics) OR TITLE (therapy) OR TITLE-ABS-KEY (treatment) OR TITLE (“drug therapy”) OR TITLE (intervention) OR TITLE (outcome*) OR TITLE (result*) OR TITLE (pharmacological) OR TITLE (trial) OR TITLE (antidepress*) OR TITLE (psychotherapy) OR TITLE (treatment) OR TITLE (psychologic*) OR TITLE (cognitive) OR TITLE (behavior) OR TITLE (controlled)) AND (TITLE (neoplasm*) OR TITLE-ABS-KEY (cancer) OR TITLE (cancer) OR TITLE (malignan*) OR TITLE (tumor) OR TITLE (tumour)) AND (TITLE-ABS-KEY (randomized OR controlled OR trial))
