# Supplementary material for: Depression Screening and Patient Outcomes in Cancer: A Systematic Review
Source: PLoS One. 2011 Nov 14;6(11):e27181. doi: 10.1371/journal.pone.0027181 (PMC3215716; doi:10.1371/journal.pone.0027181)
Supplement: Supplementary Information S2 — Relevant Systematic Reviews. (DOC) [file pone.0027181.s002.doc]

**Supplementary Information 2: Relevant Systematic Reviews**

1. Bidstrup PE, Johansen C, Mitchell AJ. Screening for cancer-related distress: Summary of evidence from tools to programmes. *Acta Oncol*. 2011;50(2):194-204.

2. Carlson LE, Clifford SK, Groff SL, Maciejewski O, Bultz BD. Screening for depression in cancer care. In: Mitchell AJ, Coyne JC, eds. *Screening for Depression in Clinical Practice.* New York: Oxford University Press; 2010:265-298.

3. Fann JR, Thomas-Rich AM, Katon WJ, et al. Major depression after breast cancer: A review of epidemiology and treatment. *Gen Hosp Psychiatry*. 2008;30(2):112-126.

4. Luckett T, Butow PN, King MT, Oguchi M, Heading G, Hackl NA, et al. A review and recommendations for optimal outcome measures of anxiety, depression and general distress in studies evaluating psychosocial interventions for English-speaking adults with heterogeneous cancer diagnoses. *Support Care Cancer*. 2010;18:1241-1262.

5. Meyer TJ, Mark MM. Effects of psychosocial interventions with adult cancer patients: A meta-analysis of randomized experiments. *Health Psychol*. 1995;14(2):101-108.

6. Mitchell AJ. Pooled results from 38 analyses of the accuracy of distress thermometer and other ultra-short methods of detecting cancer-related mood disorders. *J Clin Oncol*. 2007;25(29):4670-4681.

7. Mitchell AJ. Are one or two simple questions sufficient to detect depression in cancer and palliative care? A Bayesian meta-analysis. *Br J Cancer*. 2008;98(12):1934-1943.

8. Mitchell AJ. Short screening tools for cancer-related distress: A review and diagnostic validity meta-analysis. *J Natl Compr Canc Netw*. 2010;8(4):487-494.

9. Mitchell AJ, Meader N, Symonds P. Diagnostic validity of the hospital anxiety and depression scale (HADS) in cancer and palliative settings: A meta-analysis. *J Affect Disord*. 2010;126(3):335-348.

10. Morse R, Kendell K, Barton S. Screening for depression in people with cancer: The accuracy of the hospital anxiety and depression scale. *Clin Effect Nurs.* 2005;9(3-4):188-196.

11. Nelson CJ, Cho C, Berk AR, Holland J, Roth AJ. Are gold standard depression measures appropriate for use in geriatric cancer patients? A systematic evaluation of self-report depression instruments used with geriatric, cancer, and geriatric cancer samples. *J Clin Oncol*. 2010;28(2):348-356.

12. Ng CG, Boks MP, Zainal NZ, de Wit NJ. The prevalence and pharmacotherapy of depression in cancer patients. *J Affect Disord.* 2011;131(1-3):1-7.

13. Thekkumpurath P, Venkateswaran C, Kumar M, Bennett MI. Screening for psychological distress in palliative care: A systematic review. *J Pain Symptom Manage*. 2008;36(5):520-528.

14. Vodermaier A, Linden W, Siu C. Screening for emotional distress in cancer patients: A systematic review of assessment instruments. *J Natl Cancer Inst*. 2009;101(21):1464-1488.

15. Ziegler L, Hill K, Neilly L, Bennett MI, Higginson IJ, Murray SA, et al. Identifying psychological distress at key stages of the cancer illness trajectory: A systematic review of validated self-report measures. *J Pain Symptom Manage.* 2011;41(3):619-636.
