# Supplementary material for: Depression Screening and Patient Outcomes in Cancer: A Systematic Review
Source: PLoS One. 2011 Nov 14;6(11):e27181. doi: 10.1371/journal.pone.0027181 (PMC3215716; doi:10.1371/journal.pone.0027181)
Supplement: Supplementary Information S3 — Journals Included in Manual Searching. (DOC) [file pone.0027181.s003.doc]

**Supplementary Information 3: Journals Included in Manual Searching**

Acta Psychiatrica Scandinavica

American Journal of Medicine

American Journal of Psychiatry

Annals of Behavioral Medicine

Annals of Family Medicine

Annals of Internal Medicine

Archives of General Psychiatry

Archives of Internal Medicine

Australian and New Zealand Journal of Psychiatry

Biological Psychiatry

BMC Psychiatry

British Journal of Psychiatry

British Medical Journal

CA: A Cancer Journal for Clinicians

Cancer

Canadian Journal of Psychiatry

Canadian Medical Association Journal

Depression and Anxiety

European Psychiatry

General Hospital Psychiatry

Health Psychology

Herz

JAMA

Journal of Abnormal Psychology

Journal of Affective Disorders

Journal of Behavioral Medicine

Journal of Cancer Survivorship

Journal of Clinical Oncology

Journal of Clinical Psychiatry

Journal of Clinical Psychology

Journal of the National Comprehensive Cancer Network: JNCCN

Journal of General Internal Medicine

Journal of Psychosomatic Research

Journal of Supportive Oncology

Lancet

New England Journal of Medicine

New Zealand Medical Journal

Psychiatry Research

Psychological Assessment

Psychological Bulletin

Psychological Medicine

Psycho-oncology

Psychosomatic Medicine

Psychosomatics

Psychotherapy and Psychosomatics
