# Supplementary material for: Depression Screening and Patient Outcomes in Cancer: A Systematic Review
Source: PLoS One. 2011 Nov 14;6(11):e27181. doi: 10.1371/journal.pone.0027181 (PMC3215716; doi:10.1371/journal.pone.0027181)
Supplement: Supplementary Information S4 — Variables Included in Data Extraction Form. (DOC) [file pone.0027181.s004.doc]

**Supplementary Information 4: Variables Included in Data Extraction Form**

**Key Question #1**

Authors

Year

Country

Type of cancer / primary cancer site

Disease stage

Treatment stage

Key inclusion criteria

Key exclusion criteria

Number of patients

Recruitment rate of eligible patients

Mean age

Percent male

Structured interview used

Number (%) with major depressive disorder

Screening tool and cutoff threshold

Derivation of cutoff (e.g., literature, ROC)

Number (%) above threshold on screening tool

Interviewer access to screening results?

Number positive MDD/positive screening tool

Number positive MDD/negative screening tool

Number negative MDD/positive screening tool

Number negative MDD/negative screening tool

Sensitivity

Specificity

Positive predictive value

Negative predictive value

Notes

**Key Question #2**

Authors

Year

Country

Study funding source

Type of cancer / primary cancer site

Disease stage

Treatment stage

Key inclusion criteria

Key exclusion criteria

Mean age

Percent male

Structured interview used

Treatment

Treatment protocol (e.g., dose, hours per week)

Control group (e.g., UC, placebo, supportive therapy)

Weeks of treatment

N treatment

N control

Treatment N (%) remission

Control group N (%) remission

Response criterion

Treatment N (%) response

Control group N (%) response

For each depression outcome variable:

Outcome variable

Effect size (+ for treatment better)

Intent to treat?

N drop out / lost to follow-up

Baseline equivalence of treatment and control groups

Effect size of baseline non-equivalence (if applicable)

Blinding

Analyses adjusted/unadjusted

Primary outcome measure

Outcome measure status

Conflict of interest

Notes

**Key Question #3**

Author

Year

Country

Study funding source

Type of cancer / primary cancer site

Cancer stage

Treatment stage

Mean age

% male

Inclusion criteria

Exclusion criteria

N treatment

N control

Treatment / intervention procedure

Control condition

Screening instrument and cutoff

Method to diagnose MDD

Follow-up time

Primary outcome measure

Effect size

N drop out / lost to follow-up
