# Supplementary material for: Depression Screening and Patient Outcomes in Cancer: A Systematic Review
Source: PLoS One. 2011 Nov 14;6(11):e27181. doi: 10.1371/journal.pone.0027181 (PMC3215716; doi:10.1371/journal.pone.0027181)
Supplement: Supplementary Information S5 — Quality Assessment of Diagnostic Accuracy Studies (QUADAS) - items scored yes, no, or unclear 26 . (DOC) [file pone.0027181.s005.doc]

**Supplementary Information 5:**

**Quality Assessment of Diagnostic Accuracy Studies (QUADAS) - items scored yes, no, or unclear**^26^

| **Item** |  | **Yes** | **No** | **Unclear** |
| --- | --- | --- | --- | --- |
| 1. | Was the spectrum of patients clearly representative of the patients who will receive the test in practice? | **( )** | **( )** | **( )** |
| 2. | Were selection criteria clearly described? | **( )** | **( )** | **( )** |
| 3. | Is the reference standard likely to correctly classify the target condition? | **( )** | **( )** | **( )** |
| 4. | Is the time period between reference standard and index test short enough to be reasonably sure that the target condition did not change between the two tests? | **( )** | **( )** | **( )** |
| 5. | Did the whole sample or a random selection of the sample, receive verification using a reference standard of diagnosis? | **( )** | **( )** | **( )** |
| 6. | Did patients receive the same reference standard regardless of the index test result? | **( )** | **( )** | **( )** |
| 7. | Was the reference standard independent of the index test (i.e. the index test did not form part of the reference standard)? | **( )** | **( )** | **( )** |
| 8. | Was the execution of the index test described in sufficient detail to permit replication of the test? | **( )** | **( )** | **( )** |
| 9. | Was the execution of the reference standard described in sufficient detail to permit its replication? | **( )** | **( )** | **( )** |
| 10. | Were the index test results interpreted without knowledge of the results of the reference standard?^a^ | **( )** | **( )** | **( )** |
| 11. | Were the reference standard results interpreted without knowledge of the results of the index test? | **( )** | **( )** | **( )** |
| 12. | Were the same clinical data available when test results were interpreted as would be available when the test is used in practice?^a^ | **( )** | **( )** | **( )** |
| 13. | Were missing data on the index test handled correctly?^b^ | **( )** | **( )** | **( )** |
| 14. | Were withdrawals from the study explained? | **( )** | **( )** | **( )** |

^a^ Items #10 (blind interpretation of test results) and #12 (same clinical data available as in practice) were not evaluated because scoring of all self-report depression screening tools is fully automated and does not require judgment. ^b^ Item #13 originally was “Were uninterpretable, indeterminate or intermediate test results reported?" This item was adapted as "Were missing data on the index test handled correctly?”
