# Supplementary material for: Depression Screening and Patient Outcomes in Cancer: A Systematic Review
Source: PLoS One. 2011 Nov 14;6(11):e27181. doi: 10.1371/journal.pone.0027181 (PMC3215716; doi:10.1371/journal.pone.0027181)
Supplement: Supplementary Information S6 — The Cochrane Tool for Assessing Risk of Bias 80 . (DOC) [file pone.0027181.s006.doc]

**Supplementary Information 6: The Cochrane Tool for Assessing Risk of Bias [24]**

**Sequence generation:** Describe the method used to generate the allocation sequence in sufficient detail to allow an assessment of whether it should produce comparable groups.

**Allocation concealment** Describe the method used to conceal the allocation sequence in sufficient detail to determine whether intervention allocations could have been foreseen in advance of, or during, enrolment.

**Blinding of participants, personnel and outcome assessors** *Assessments should be made for each main outcome (or class of outcomes).* Describe all measures used, if any, to blind study participants and personnel from knowledge of which intervention a participant received. Provide any information

relating to whether the intended blinding was effective.

**Incomplete outcome data** *Assessments should be made for each main outcome (or class of outcomes).* Describe the completeness of outcome data for each main outcome, including attrition and exclusions from the analysis. State whether attrition and exclusions were reported, the numbers in each intervention group (compared with total randomized participants), reasons for attrition/exclusions where reported, and any re-inclusions in analyses performed by the review authors.

**Selective outcome reporting** State how the possibility of selective outcome reporting was examined by the review authors, and what was found.

**Other sources of bias** State any important concerns about bias not addressed in the other domains in the tool. If particular questions/entries were pre-specified in the review’s protocol, responses should be provided for each question/entry. Was the study apparently free of other problems that could

put it at a high risk of bias?
