# Supplementary material for: Depression Screening and Patient Outcomes in Cancer: A Systematic Review
Source: PLoS One. 2011 Nov 14;6(11):e27181. doi: 10.1371/journal.pone.0027181 (PMC3215716; doi:10.1371/journal.pone.0027181)
Supplement: Table S1 — Excluded Studies for Effect of Screening on Depression Outcomes (Key Question #3). (DOCX) [file pone.0027181.s007.docx]

**Table S1: Excluded Studies for Effect of Screening on Depression Outcomes (Key Question #3)**

| **First Author,**  **Year,**  **Country** | **Cancer Site** | **N Consented/ Randomized^a^** | **Comparison** | **Depression Outcomes** | **Reason(s) for exclusion** |
| --- | --- | --- | --- | --- | --- |
| Boyes [S1],  2006,  Australia | Mixed | 80 | **Intervention**: Results from a computer survey completed prior to each visit were provided to the patient’s oncologist. The survey included an assessment of 12 physical symptoms associated with chemotherapy, symptoms of anxiety and depression (HADS), and perceived supportive care needs (31 items), along with computer-generated suggested strategies to manage each identified issue.  **Control**: Results from computer survey not made available to oncologist. | No significant difference after 4 visits between groups for change in HADS-D scores and proportion of patients with HADS-D ≥ 11. | Screening of multiple problems and perceived care needs did not allow assessment of the effect of depression screening. |
| Carlson [S2],  2010,  Canada | Lung and breast | 1,134 | **Full Screening Intervention**: Results from DT, problem checklist, fatigue and pain thermometers, and PSSCAN depression and anxiety sections, along with personalized feedback report placed on patient’s electronic medical record at initial visit.  **Triage Intervention:** Full screening, as described above, along with an offer to speak to a member of the study psychosocial team about any of the assessed issues.  **Control**: DT completed, but results were not disclosed to patient or placed on electronic medical record. | No difference between full screening intervention, triage intervention, or usual care groups on PSSCAN depression scores 3 months post-randomization. | Screening of multiple problems did not allow assessment of the effect of depression screening. |
| Detmar [S3],  2002,  Netherlands | Mixed | 273^b^ | **Intervention**: Patients completed a quality of life questionnaire, the EORTC QLQ-C30, at 3 successive outpatient visits with results made available to patient and physician prior to consultation.  **Control:** Usual care. | No difference between groups in SF-36 Mental Health subscale after 4^th^ visit. | A positive depression screen based on a defined cutoff score was not used to determine who received further assessment or treatment. |
| Hilarius [S4],  2008,  Netherlands | Mixed | 298 | **Intervention:** Patients completed a quality of life questionnaire, the EORTC QLQ-C30, at 4 outpatient visits, with summaries given to patients and nurses prior to consultation.  **Control:** Standard consultations with physicians and nurses. | No difference between groups in SF-36 Mental Health subscale after 4^th^ visit. | Not a randomized controlled trial (sequential cohort design). In addition, a positive depression screen based on a defined cutoff score was not used to determine who received further assessment or treatment. |
| Maunsell [S5],  1996  Canada | Breast | 261 | **Intervention:** Usual care + telephone screening beginning 21 days post-randomization and repeated at 28-day intervals with the 20-item GHQ. Social workers made follow-up telephone calls to all patients with GHQ ≥ 5 to assess psychological distress and offer psychosocial intervention.  **Control**: Usual care, which included minimal psychosocial intervention as part of initial cancer care. | Distress levels on the PSI decreased for both groups. No between-group differences at 3 and 12 months. | Screening was for distress, broadly, not MDD and the follow-up intervention for positive screens was geared towards distress, not MDD specifically.. |
| McLachlan [S6],  2001,  Australia | Mixed | 450 | **Intervention:** Patients at their first consultation completed a series of self-report questionnaires via touch-screen computer, including the CNQ, EORTC QLQ-C30, and BDI-SF. A summary of questionnaire results was made available to physicians prior to consultation, which were intended to be used to inform an individualized management plan.  **Control:** Questionnaire responses were not made available to health care team prior to consultation. | No significant difference between groups at 2-months or 6-months post-randomization for the CNQ psychologic domain, BDI-SF scores or EORTC QLQ-C30 Emotional Functioning.^c^ | A positive depression screen based on a defined cutoff score was not used to determine who received further assessment or treatment. Additionally, screening of multiple problems did not allow assessment of the effect of depression screening. |
| Rosenbloom [S7],  2007,  USA | Mixed | 213 | **Assessment, Interview, and Discussion Intervention:** At baseline, 1 and 2 months, patients completed FLIC and FACT-G, and FACT-G scores elaborated through an interview and discussion, the results of which were shared with treatment nurse prior to visit.  **Assessment Intervention:** At baseline, 1 and 2 months, patients completed FLIC and FACT-G, and FACT-G scores were shared with treatment nurse prior to visit.  **Control:** Patients completed FLIC at baseline. Questionnaire data not shared with treatment nurse. | No significant difference between groups at 3-months or 6-months post-randomization for Brief POMS negative mood subscale or FLIC psychological subscale scores. | A positive depression screen based on a defined cutoff score was not used to determine who received further assessment or treatment. Additionally, screening of multiple problems did not allow assessment of effect of depression screening. |
| Shimizu [S8],  2010,  Japan | Mixed^d^ | 1,065 | **Intervention:** Patients completed 11-point DIT (score range 0-10), and those with a distress score ≥ 4 and an impact score ≥ 3 were referred by their oncologist for a psycho-oncology service consultation.  **Control:** Usual care with referral to psycho-oncology services by physician if considered moderately or severely distressed. | Only number of positive screens and number diagnosed and treated, but no depression outcomes, were assessed. | Not a randomized controlled trial (sequential cohort design). In addition, outcomes included number of positive screens and number treated, but no depression outcomes were assessed. |
| Taenzer [S9],  2000,  Canada | Lung | 57 | **Intervention:** At a single clinic visit, patients completed the EORTC QLQ-C30, which was provided to clinic staff prior to clinic appointment with no specific instructions for use.  **Control**: Patients completed the EORTC QLQ-C30 following their clinic appointment. | Only number of quality of life issues addressed in appointment and patient satisfaction, but no depression outcomes, were assessed. | Not a randomized controlled trial (sequential cohort design). In addition, a positive depression screen based on a defined cutoff score was not used to determine who received further assessment or treatment and no depression outcomes were assessed. |
| Thewes [S10],  2009,  Australia | Mixed | 83 | **Intervention:** Patients completed the DT, and nursing staff was encouraged to assess problems and discuss psychosocial referral for patients with DT score ≥ 5.  **Control:** Usual care with no screening. | Contrary to hypothesis, patients in the screened group reported significantly higher level of unmet psychological needs 6 months after initial clinic contact. | Not a randomized controlled trial (sequential cohort design). Outcome of unmet psychological needs, but not depression. |
| Velikova [S11],  2004,  UK | Mixed | 286 | **Assessment and Feedback Intervention:** For 6 month study period, prior to clinic visits, patients completed EORTC QLQ-C30 and HADS with results provided to physicians prior to visit.  **Attention Control:** For 6 month study period, prior to clinic visits, patients completed EORTC QLQ-C30 and HADS with no results provided to physicians.  **Usual Care Control:** Patients did not complete EORTC QLQ-C30 or HADS. | Scores on FACT-emotional subscale were better in the intervention group than the usual care control group, but not different from the attention control group. | A positive depression screen based on a defined cutoff score was not used to determine who received further assessment or treatment. Additionally, screening of multiple problems did not allow assessment of effect of depression screening. |

Abbreviations: DT = Distress Thermometer; BDI-SF = Beck Depression Inventory – Short Form; CNQ = Cancer Needs Questionnaire; DIT = Distress and Impact Thermometer; EORTC QLQ-C30 = European Organization for Research and Treatment of Cancer Quality of Life Questionnaire - Core 30; FACT-emotional = Functional Assessment of Cancer Therapy-emotional; FACT-G = Functional Assessment of Cancer Therapy-General; FLIC = Functional Living Index – Cancer; GHQ = General Health Questionnaire; HADS = Hospital Anxiety and Depression Scale; HADS-D = Depression subscale of Hospital Anxiety and Depression Scale; MDD = Major depressive disorder; POMS = Profile of Mood States; PSI = Psychiatric Symptom Index; PSSCAN = Psychological Screen for Cancer; SF-36 = Short Form 36 Health Survey Questionnaire.

^a^ Number consented for non-randomized controlled trials and number randomized for randomized controlled trials. ^b^ Physicians, rather than participants, were randomized. This number is the number of eligible patients who agreed to participate. ^c^ The authors reported a post-hoc subgroup analysis that found significantly improved BDI-SF scores for the 44 patients in the intervention group with baseline BDI-SF scores ≥ 8 compared to the 19 control patients with BDI-SF ≥ 8. However, patients were not randomized based on BDI-SF scores, and the relevance of these results for screening are not clear, since screening is applied to all patients, not only patients identified through screening with high scores. ^d^ 95% of patients were female.

**REFERENCES FOR TABLE S1**

S1. Boyes A, Newell S, Girgis A, McElduff P, Sanson-Fisher R (2006) Does routine assessment and real-time feedback improve cancer patients' psychosocial well-being? Eur J Cancer Care 15: 163-71.

S2. Carlson LE, Groff SL, Maciejewski O, Bultz BD (2010) Screening for distress in lung and breast cancer outpatients: a randomized controlled trial. J Clin Oncol 28: 4884-91.

S3. Detmar SB, Muller MJ, Schornagel JH, Wever LD, Aaronson NK (2002) Health-related quality-of-life assessments and patient-physician communication: a randomized controlled trial. JAMA 288: 3027-34.

S4. Hilarius DL, Kloeg PH, Gundy CM, Aaronson NK (2008) Use of health-related quality-of-life assessments in daily clinical oncology nursing practice: a community hospital-based intervention study. Cancer 113: 628-37.

S5. Maunsell E, Brisson J, Deschenes L, Frasure-Smith N (1996) Randomized trial of a psychologic distress screening program after breast cancer: effects on quality of life. J Clin Oncol 14: 2747-55.

S6. McLachlan SA, Allenby A, Matthews J, Wirth A, Kissane D, et al. (2001) Randomized trial of coordinated psychosocial interventions based on patient self-assessments versus standard care to improve the psychosocial functioning of patients with cancer. J Clin Oncol 19: 4117-25.

S7. Rosenbloom SK, Victorson DE, Hahn EA, Peterman AH, Cella D (2007) Assessment is not enough: a randomized controlled trial of the effects of HRQL assessment on quality of life and satisfaction in oncology clinical practice. Psychooncology 16: 1069-79.

S8. Shimizu K, Ishibash Y, Umezawa S, Izumi H, Akizuki N, et al. (2010) Feasibility and usefulness of the 'Distress Screening Program in Ambulatory Care' in clinical oncology practice. Psychooncology 19: 718-25.

S9. Taenzer P, Bultz BD, Carlson LE, Speca M, DeGagne T, et al. (2000) Impact of computerized quality of life screening on physician behaviour and patient satisfaction in lung cancer outpatients. Psychooncology 9: 203-13.

S10. Thewes B, Butow P, Stuart-Harris R (2009) Greater So Area Hth Serv Screening. Does routine psychological screening of newly diagnosed rural cancer patients lead to better patient outcomes? Results of a pilot study. Aust J Rural Health 17: 298-304.

S11. Velikova G, Booth L, Smith AB, Brown PM, Lynch P, et al. (2004) Measuring quality of life in routine oncology practice improves communication and patient well-being: a randomized controlled trial. Journal of Clinical Oncology 22: 714-24.
